# Case report: Imaging of septic arthritis in the hip joint of a calf treated with femoral head ostectomy

**DOI:** 10.3389/fvets.2024.1292924

**Published:** 2024-07-18

**Authors:** Takeshi Tsuka, Yoshiharu Okamoto, Atsushi Nishiyama, Yuji Sunden, Takehito Morita

**Affiliations:** ^1^Department of Veterinary Clinical Medicine, Joint Department of Veterinary Medicine, Faculty of Agriculture, Tottori University, Tottori, Japan; ^2^WOLVES HAND Advanced Veterinary Medical Research Institute, Osaka, Japan; ^3^Okayama Prefectural Federation Agricultural Mutual Aid Association, Okayama, Japan

**Keywords:** calf, computed tomography, femoral head ostectomy, hip arthritis, ultrasonography

## Abstract

A 24-day-old male Japanese Black calf presented with weight-bearing lameness in the left hind limb and a swollen pelvis. Ultrasonography revealed the accumulation of fluidity materials with a mixture of hyper- and hypo-echogenicity, enclosed by 5–10-mm-thick capsular structures. On the ventral-dorsal radiograph of the pelvis, irregular and radiolucent osseous changes were evident in the femoral head and acetabulum within the swollen hip joint, with soft-tissue density. Computed tomography (CT) confirmed the relationship between the bony and soft tissue lesions, which was suspected following ultrasonography and radiography, and provided additional findings, such as intra-articular accumulation of gas and the degree of osteolytic changes. Based on the imaging findings and cytology of the arthrocentesis specimen, the patient was diagnosed with hip arthritis and osteomyelitis of the femoral head. Additionally, the severity of the lesions supported our decision to perform a femoral head ostectomy. The postoperative radiographic and CT findings of the treated pelvis were helpful in evaluating the efficacy of this technique. Following treatment, the animal could walk and showed normal development, although it was three postoperative months before weight-bearing lameness improved. To the best of our knowledge, this is the first report to describe the combined use of ultrasonography, radiography, and CT in the diagnosis, preoperative planning, and evaluation of the postoperative effects of bovine hip arthritis. Additionally, this report details the therapeutic efficacy of femoral head ostectomy for bovine hip arthritis, a technique that has not been reported previously.

## Introduction

Hip lameness is characterized by abnormal postures, including asymmetry in the heights of the greater trochanter and pelvis (tuber ischii), and abnormal walking with a rolling outward motion and short stride in the affected hind limb, if the animal can stand (1, 2). It is required to differentiate between coxofemoral luxation, fractures of the femoral head/neck and acetabulum, slipped capital femoral epiphysis, and hip dysplasia as orthopedic diseases in newborn and younger calves when exhibiting these clinical signs (1–3). Septic arthritis of the hip joints (hip arthritis) is one of these diseases, which accounted for 10% of animals with hip lameness (4). The suspicion of these diseases can be confirmed by investigating the anatomical location, such as the pelvis and greater trochanter, in which the crepitus can be palpated, as well as by observing the appearance and severity of pain induced during manipulation of the affected limb during flexion, extension, or swing (1). Swelling of the hip joints is a significant sign, together with fluctuations and hardness on palpation (1, 5, 6). However, bovine hip arthritis is not always associated with swollen hip (5, 6). Macroscopic and physical examinations can provide suggestive but not diagnostic evidence because of the similarities in findings among these hip diseases (1, 2, 6).

Radiography is a commonly used imaging modality for diagnosis, subsequent treatment options, and final judgment of prognosis (1). However, radiography tends to be less convenient because of technical limitations such as the forced restraint of the dorsal position required to take a valuable ventral-dorsal (VD) radiograph, the need for sedation or general anesthesia, and the poor quality of radiographs taken from large-sized animals (2, 7, 8). Additionally, radiological waves of powerful magnitude are required to penetrate a thick body mass (9). Ultrasonography is applicable for the observation of bovine hip joints by percutaneous or transrectal scanning and has previously been used to diagnose coxofemoral luxation and hip arthritis in bovines (2, 5, 9, 10). However, a transducer with a lower ultrasound frequency is required to observe the hip joints lying at 12 of 18 cm depth from the skin in adult cattle, which produces a lower-resolution image (9).

The utility of advanced imaging modalities, such as computed tomography (CT) and magnetic resonance imaging, has recently been indicated in bovine orthopedics, despite the requirement for general anesthesia during examination (3, 9, 11). CT can provide three-dimensional (3D) reconstruction images to visualize various osseous lesions such as osteomyelitis (12, 13). Pelvic CT has been used for the morphometric evaluation of specimens from slaughtered cattle (14). However, to our knowledge, there have been no reports describing the CT evaluation of bovine hip arthritis despite the high diagnostic efficacy of CT in human medicine (15, 16).

The main purpose of this report was to explain the efficacy of a combination of ultrasonography, radiography, and CT in the preoperative diagnosis of extensive hip arthritis in a Japanese Black calf, and to evaluate the success of surgery when the animal was examined soon after femoral head ostectomy (FHO). Additionally, we aimed to introduce the technical procedures and outcomes of FHO. We discuss our results along with previous clinical data from therapy and imaging studies in human and veterinary medicine.

## Case presentation

A 24-day-old male Japanese Black calf presented with lameness in the left hind limb that was observed immediately after birth. This clinical sign did not improve with continuous administration of antibiotics (cefazolin injection, Fujita Pharmaceutical Co., Ltd., Tokyo, Japan). Swelling gradually developed at the left hip joint. The animal was admitted to our veterinary hospital with suspected coxofemoral luxation. On admission, the animal showed severe weight-bearing lameness, with knuckling of the left hind limb. Based on criteria described by Desrochers et al., the lameness score was 2 (17). An elevated white blood cell (WBC) count (23,100/μl; reference value: 6,500–11,500/μl) was evident on hematological examination using a hematology automatic analyzer (pocH-100iV Diff; Sysmex Co., Ltd., Hyogo, Japan) (18).

**Figure 1:**
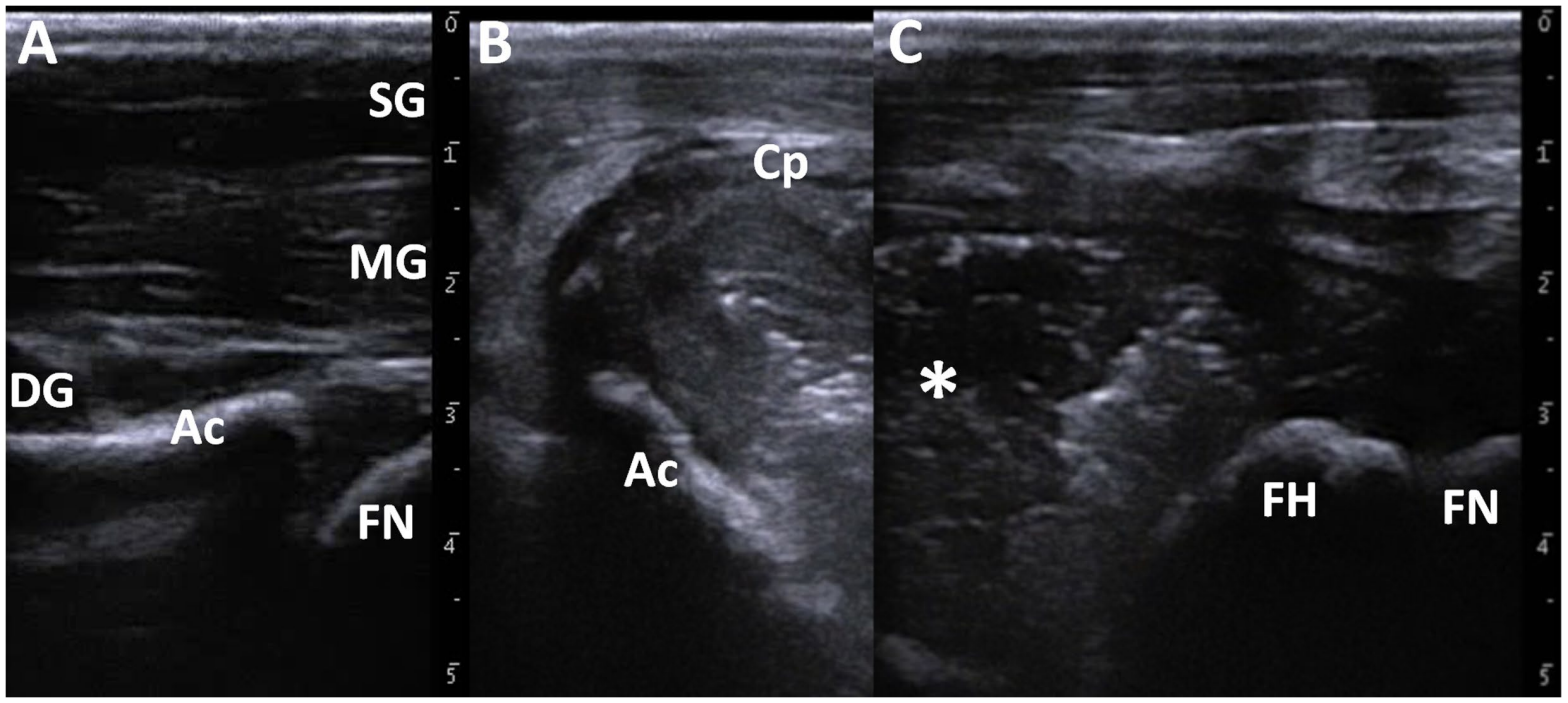
Ultrasonographic images of the healthy right **(A)** and affected left hip joints **(B,C)**. **(A)** Echogenic lines of the femoral neck (FN) and the acetabulum (Ac) are evident at a deeper site than the pelvic muscular layers comprising of the superficial gluteus muscle (SG), the middle gluteal muscle (MG), and the deep gluteal muscle (DG). **(B)** When scanning near the pelvis, the 5–10-mm-thick wall of the capsule (Cp) encloses the fluidity materials with a mixture of hyper- and hypo-echogenicity. **(C)** When scanning around the area of the femur, the round, echogenic surface of the femoral head (FH) and femoral neck (FN) are evident within the capsular lesion including the fluidity materials (asterisk).

A portable ultrasound machine (MyLabOne VET; Esaote Co., Genova, Italy) was used to observe the articular cavities of both hip joints in the standing position without sedation. A 10 MHz linear transducer was applied to the dorsal hip surface near the pelvis and subsequently moved toward the greater trochanter. Ultrasonography of the right hip joint revealed normal appearance of the smooth echogenic line of the femoral neck and the echogenic line of the acetabulum (Figure 1A). The structure of the femoral head was distally unclear from that of the acetabulum. On the ultrasonogram of the left hip joint, 5–10-mm-thick capsular structures were present in the region 1–2 cm deeper than the skin surface and across the anatomical level between the pelvis and femoral head (Figures 1B,C). The walls were lined with striped structures that appeared slightly hypoechoic compared to the superficial muscular structures. The end structures of the walls could be observed by curving toward the deeper region near the acetabulum, although the end structures were unclear at the level of the femur. The capsular walls enclosed the fluid material and appeared as a mixture of hyperechoic and hypoechoic contents. Acoustic shadowing was not observed within the cavity. The femoral head was a smooth, hyperechoic line running at a depth of approximately 3 cm within the cavity. Osteolytic changes were not observed on the femoral surface. Under ultrasonographic imaging, 30 mL of the synovial fluid was aspirated using a 22-gage needle (Terumo Spinal Needle, Terumo Co., Tokyo, Japan). The aspirate was a cream-colored turbid fluid with low viscosity. The synovial WBC count was 15,000 cells/μl (reference value: 135–163 cells/μl) (19). Cytology of the synovial fluid revealed that 99% of the cell components were neutrophils (reference value: 7.6 ± 10.7%), together with small masses of bacteria (19). Bacterial examination revealed the isolation of group B *Streptococcus* from the synovial fluid.

**Figure 2:**
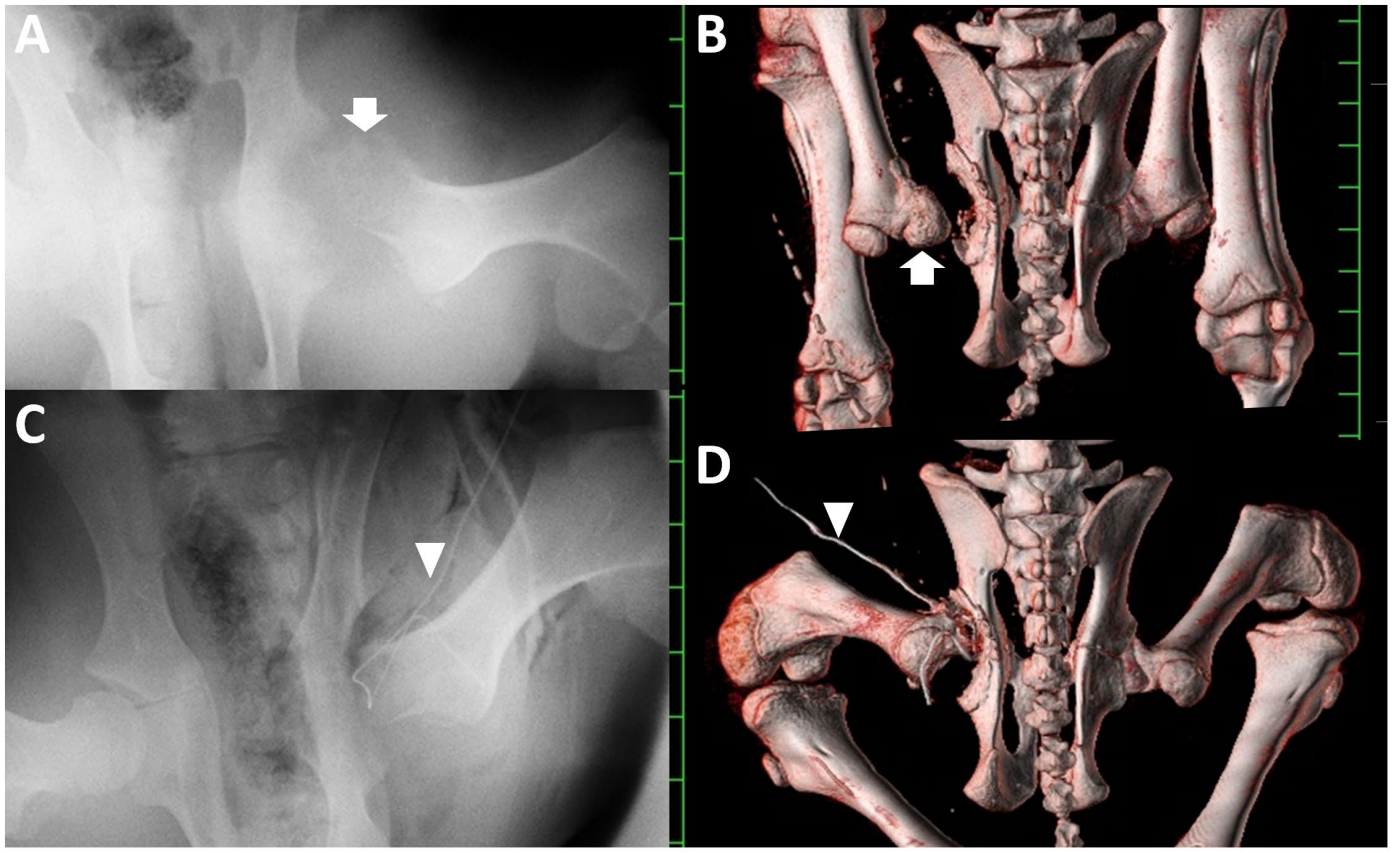
Ventral-dorsal pelvic radiographs and three-dimensional computed tomographic images of the dorsal skeletal views of the pelvis taken at admission (**A,B**, respectively) and soon after surgery (**C,D**, respectively). **(A)** An irregular, radiolucent femoral head (arrow) is evident within the swollen hip joint. **(B)** Small bone fragments are present within the extended gap between the transformed femoral head (arrow) and the irregular surface of the acetabulum. **(C)** The irregular cut surface of the femur penetrates the irregular cup of the acetabulum. **(D)** The cut surface of the femoral neck is located almost inside the irregular cup of the acetabulum. Arrowhead: a Penrose drain. Scale: 25 mm.

A computed radiography machine (REGIUS Console CS-3, Konica Minolta Health Care, Japan) was used to observe the hip joints of the animal anesthetized with 2–3% isoflurane via an endotracheal tube inserted following sedation with an intravenous injection of xylazine hydrochloride (0.2 mg/kg). VD radiographs with the animal’s hind limb in the supine position revealed that the articular gap between the acetabulum and the femoral head was extended (Figure 2A). The extended gap had a homogeneous soft-tissue density, in which the radiodensity was slightly lower than that of the peripheral swollen muscular structures of the left hip joint. The femoral head was seen as a heterogeneous radiolucent structure compared to the shaft of the femur. Additionally, irregular and heterogeneous radiolucent osseous changes were observed on the acetabular surface.

**Figure 3:**
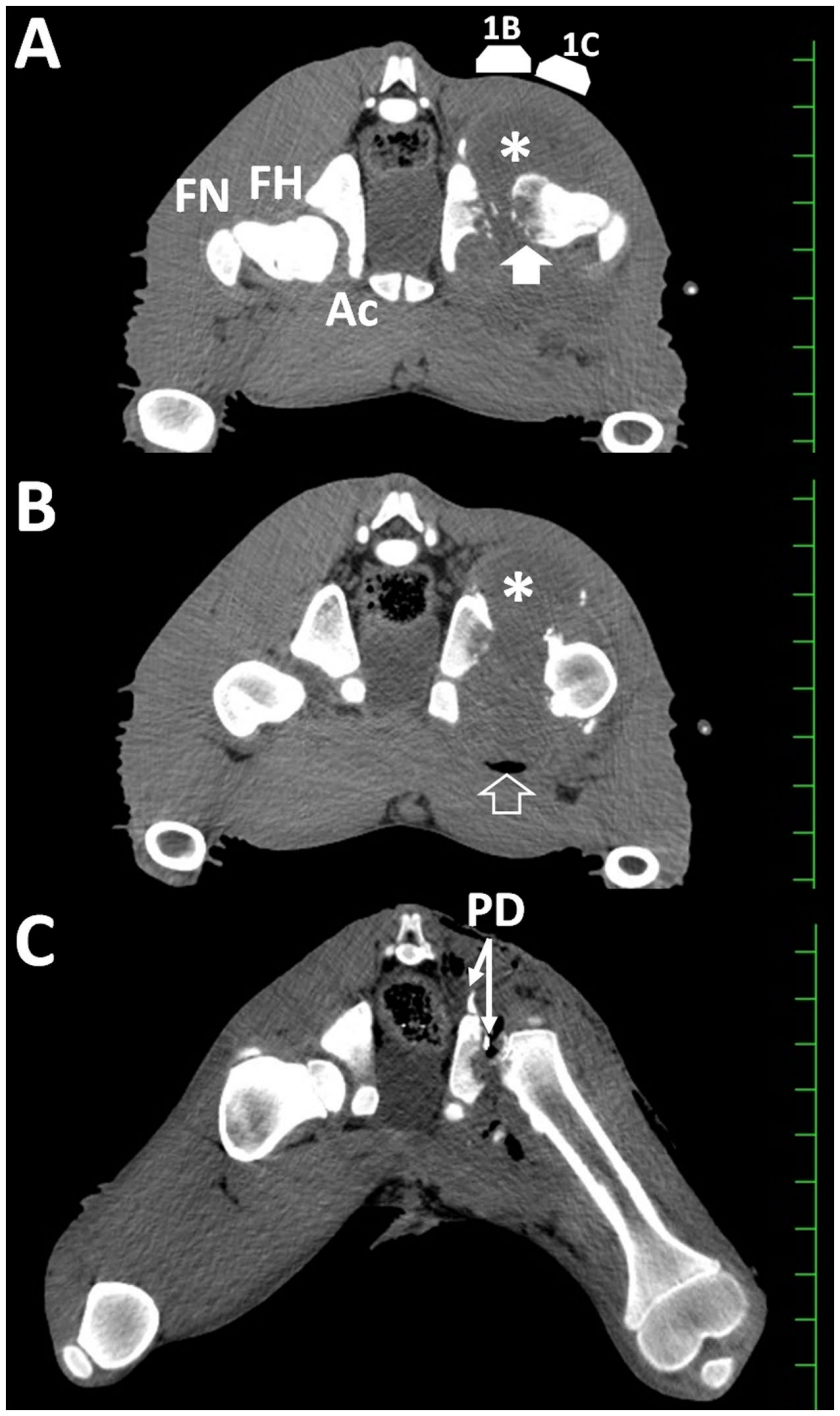
Transverse computed tomographic images taken at admission **(A,B)** and soon after surgery **(C)**. **(A)** Small bone fragments are present between the heterogeneously hypoattenuating femoral head (FH, arrow) and the irregular surface of the acetabulum (Ac) within the extended, hypoattenuating capsular mass (asterisk). 1B and 1C show the scanning locations of Figures 1B,C, respectively. HN, femoral neck. **(B)** Accumulation of gas (arrowhead) is evident within the capsular mass (asterisk). **(C)** The gap between the irregular surfaces of the acetabulum and cut surface of the femur is narrower than those shown in **(A)**. PD: a Penrose drain. Scale: 25 mm.

Soon after the radiographic examination (under the same anesthesia), the animal was examined in the supine position using a 16-section multidetector scanner (ECLOS, Hitachi Co. Ltd., Tokyo, Japan). Transverse CT showed that the hip joint lesion extended significantly in the circumferential direction from the central femoral head (Figure 3A). The extended hip lesion comprised heterogeneously hypoattenuating structures with smooth dorsal and irregular ventral surfaces, outlined by a distinct border on the peripheral soft tissue structures of the left hind limb. The VD length of the lesion was approximately 10 cm in length on CT images. Small hyperattenuating spots were evident within the extending gap between the acetabulum and femoral head, suggesting the presence of multiple bone fragments. The width in the gap between the acetabulum and femoral head was >20 mm. The femoral head appeared partly as a smooth, curved line on the dorsal surface, and mostly as intermittent lines on the ventral surface, indicative of osteolytic changes. The internal region of the femoral head was heterogeneously hypodense compared with that of the femoral neck. Articulation and bony structures were normal in the right hip joint. On transverse CT image slightly caudal to the section shown in Figure 3A, a crescentic accumulation of air was evident along the ventral margin of the lesion because the air moved to the upper side of the lesion when examined in a supine position (Figure 3B). The 3D CT images were obtained using an image analysis system (AZE Virtual Place; AZE Corp., Tokyo, Japan). A 3D CT image of the dorsal side of the pelvis showed that the femoral head had an irregular and transformed surface on the left hind limb (Figure 2B). The articular gap was extended and included small bone fragments between the femoral head and acetabulum. The left acetabulum had an irregular rounded surface along the extended gap, in which bone proliferation had developed dorsally. Based on the diagnostic results, arthrotomy and complete removal of the affected femoral head were recommended.

**Figure 4:**
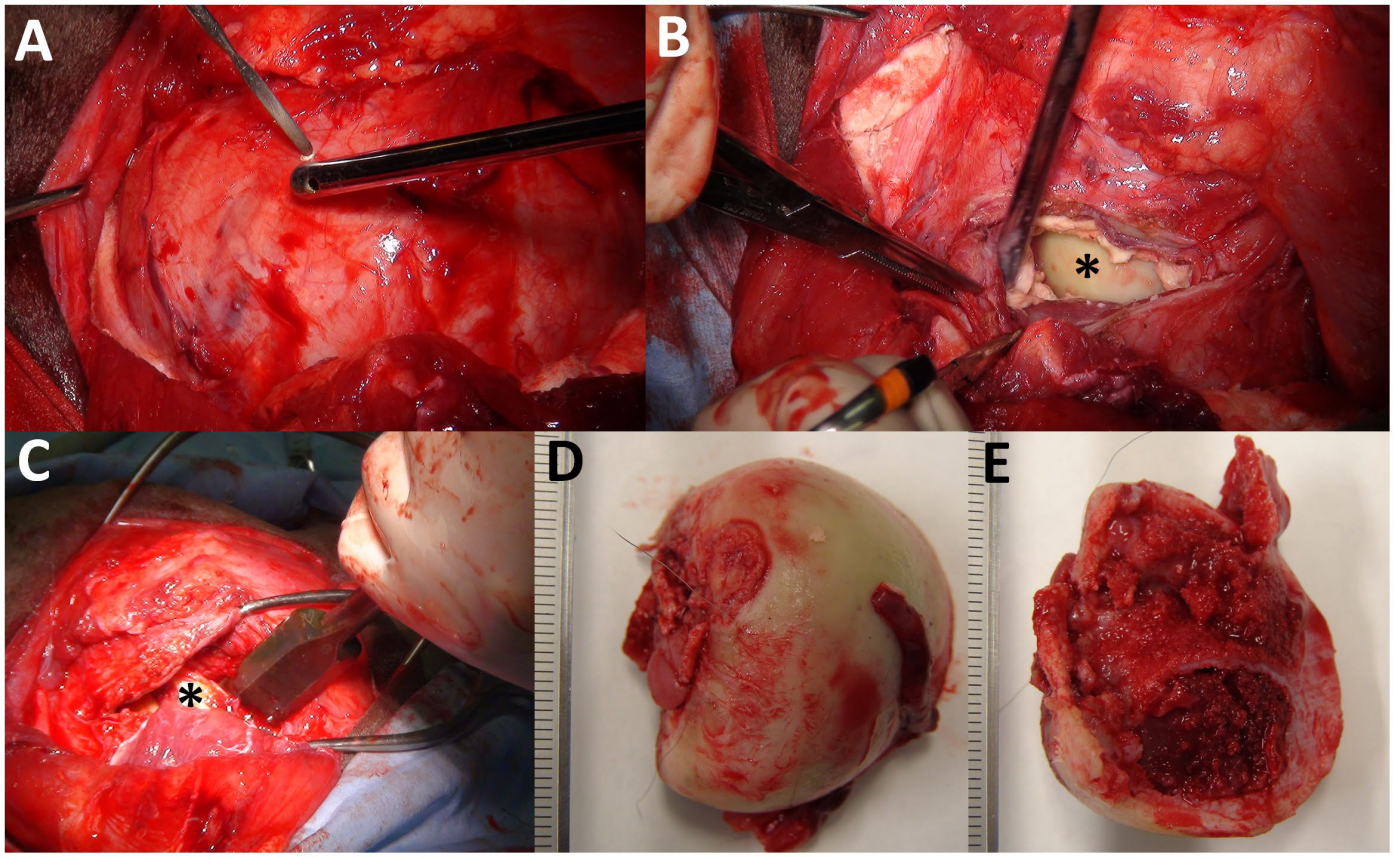
Intraoperative photos of femoral head ostectomy for septic arthritis of the hip joints together with osteomyelitis of the femoral head **(A–C)** and macroscopic views of the resected femoral head **(D,E)**. **(A)** A small cut is made on the surface of the capsular mass extending into the entire area deeper than the transected middle gluteal muscle. **(B)** The surface of the femoral head (asterisk) is partially evident inside the lesion’s cavity filled with granulation tissue. **(C)** Resection is made using a bone chisel applied to the area of the femoral neck at the farthest point possible to the side of the femoral head (asterisk). **(D)** An ulcerative lesion (arrow) is evident on the articular surface of the resected femoral head. **(E)** Discoloration is evident in the cut surface of the resected femoral head.

One day after the examination, the animal was positioned in lateral recumbency with the left hind limb uppermost while maintained under anesthesia using the above-mentioned anesthetic method. A 30-cm curved skin incision was made above the swollen region of the left hip joint. The middle gluteal muscle was exposed by blunt dissection of the subcutaneous structures and subsequently transected completely. The surface of the lesion was exposed in the entire region distal to the transected middle gluteal muscle (Figure 4A). A small cut was made in the middle of the lesion wall using cautery. Most purulent fluids were removed using a suction tube inserted through a small hole. The sciatic nerve, which lay caudal to the swollen lesion, was held with a nylon thread after blunt dissection from neighboring tissues. Segmentation of the walls of the lesion was performed using cautery as the small hole spread, followed by gradual resection to expose the cavity. After removing the caseating tissue, the femoral head was identified on the far side of the cavity (Figure 4B). The femoral head was removed by resecting the area of the femoral neck using a bone chisel (Figure 4C). The cut surface was debrided using a bone rongeur to remove pathological tissues. Debridement was further performed on the brittle and irregular surfaces of the acetabulum. Irrigation was also performed inside the cavity several times using a large amount of ozonated water provided by an ozonated water generator (Sakuragawa Pump Co., Ltd., Osaka, Japan). A Penrose drain (outer diameter, 6.0 mm; Fuji Systems Co., Fukushima, Japan) was placed into the cavity via a skin incision made at the side of the surgical wound. The lesion cavity was closed by simple interrupted suturing of the remaining capsules using an absorbable suture material (MAXON; Davis & Geck, United States). The proximal cut surface of the middle gluteal muscles was fixed to the more distal region of the biceps femoris muscle using an absorbable suture material to forcibly stabilize the remaining femoral neck into the acetabulum and cover the sutured joint capsule with the muscles. The skin was sutured using a nylon suture material (Suprylon 3–0, Vömel, Germany), and the drainage tube was secured using Chinese finger trap suturing.

An irregular cut surface was observed in the femoral neck on the VD radiograph obtained immediately after surgery (Figure 2C). Most femoral cut surfaces touched the acetabulum. On postoperative transverse CT, small amounts of exudate were present, together with scattered air accumulation within the narrow space between the irregular surface of the acetabulum and the cut surface of the femoral neck (Figure 3C). The width in the apace was measured as 10 to 15 mm. Subsequent 3D CT revealed that the femoral head could be resected at the base level, while most of the femoral neck remained intact (Figure 2D). Debris from the femoral head remained on the cut femoral surface. The cut surface of the femur is located close to the irregular cup of the acetabulum.

Antibiotics (cefazolin sodium injection; Nichi-Iko Pharmaceutical Co., Ltd., Toyama, Japan) were administered intravenously for 10 days after surgery. The sangineous exudates were continuously eliminated via a Penrose drain and were observed to have gradually decreased by the fourth postoperative day. The Penrose drain was removed on the fifth postoperative day. Weight-bearing lameness persisted in the left hind limb for the first few weeks after surgery and gradually improved between one and three months after surgery. Finally, the animal had the lameness score of 0 (17), followed by the normal development without recurrence of septic arthritis in the left hip joint.

An ulcerative lesion was macroscopically evident near the resected area of the articular surface of the femoral head (Figure 4D). Discoloration and structural fragility were further observed on the cut surface of the femoral head (Figure 4E). Histology of the removed femoral head and acetabulum revealed common findings such as aggregation of inflammatory cells and fibroblasts together with scattered formation of small abscess foci in the bone marrow tissues, osteolytic and necrotic changes in the cancellous bone layers, and infiltration of neutrophils and bacterial colonization in the bone cortex layers (Supplementary Figures S1, S2). The histology of the removed lesion walls revealed predominant proliferation of collagen fibers and granulation tissue (Supplementary Figure S3). Based on these histological findings, the animal was diagnosed with necrotizing purulent osteoarthritis, osteomyelitis, and septic arthritis.

## Discussion

In this case, pelvic radiography revealed an enlarged area of the hip joint with increasing opacity, an irregular and radiolucent femoral head, and an irregular acetabular surface. The radiographic findings indicated that the hip arthritis in this case may not have been at an early stage, because the development of osteolysis shown on radiography was evidence of chronic septic arthritis (6). However, differentiating between hip arthritis and other hip diseases can be confusing because of the similarity in the radiographs of osseous changes in the adjacent bones, which depend on a variety of primary lesions. Coxofemoral luxation and hip osteoarthritis can also cause osteophyte production in the acetabulum and femoral head (3, 10, 20). Hip joint instability associated with primary hip diseases such as hip arthritis, coxofemoral luxation, and hip dysplasia can promote the remodeling of bone structures, differentiating among these diseases at clinical examination (20).

On pelvic ultrasonogram, the accumulation of purulent exudates within the hypertrophic joint capsules was represented by heterogeneous echogenic materials with scattered hyperechoic spots enclosed by hypoechoic fibrous structures. Ultrasonography is superior to radiography for observing the pathological conditions of soft tissue structures, such as joints, tendon sheaths, and muscles, induced by septic arthritis, despite its inferior function in the evaluation of bone lesions (6, 9, 21). Joint inflammation can be suspected from visible synovial effusion and distension of the synovial pouch on the ultrasonogram of bovine joints, for which the synovial content is less evident within the healthy joint cavity (9). However, ultrasonography did not reveal any osteolytic changes in the femoral head when the transducer was placed on the dorsal surface of the swollen hip joint. This may have been associated with the unequal formation of osteolytic changes in the femoral head. Using the same scanning method, these lesions were successfully visualized as a completely irregular surface of the femoral head within the distended anechoic joint cavity, indicating hip arthritis (9).

CT is superior to radiography for evaluating the pathogenic relationship between soft tissues and osseous lesions (15). In this case, destruction of the femoral head and acetabulum was evident on CT, together with the formation of small bone fragments within the rounded area of the soft tissue mass, indicating the concurrent development of hip arthritis and osteomyelitis of these bones. Additionally, CT can identify intra-articular accumulation of gas, indicating hip arthritis associated with infection by gas-producing bacteria (6), which is evidence that could not have been obtained by radiography or ultrasonography. CT can provide evidence of osteomyelitis as a hypoattenuating region discernible from adjacent hyperattenuating bone structures (12, 13, 15). 3D reconstruction from CT was effectively used to evaluate the morphology of the osseous lesions, including the production of bone fragments, depth and location of the osteolytic defects of the femoral head, and the degree of dislocation of the femoral head (12, 13). The infectious lesions spread extensively once septic arthritis develops, resulting in mixtures of soft tissue lesions, such as abscessation and granulation, osteomyelitis, and pathological fractures in the adjacent bones (4, 5, 22, 23). This extensive pattern was accurately demonstrated by the CT images in this case. The concurrent abnormal CT appearances of soft tissues and bones resemble the characteristics of the chronic phases of hip arthritis in humans (15). It has further been suggested that medical treatments and joint lavage via arthrocentesis may not have a good therapeutic effect on arthritic joints if presented with concurrent lesions (6). Based on Verschooten’s criteria for bovine bone infections, this case was classified as a Type-2 lesion (primary osteomyelitis and secondary arthritis) or a Type-3 lesion (primary arthritis and secondary osteomyelitis) (24). However, it is usually difficult to determine the origin of infection (22, 23). Thus, it may be necessary to treat extensive bovine septic arthritis with radical arthrotomy to remove all tissues suspected as the source of infection, including the capsule, cartilage, muscle, tendon, and bone, together with joint lavage and removal of inflammatory products. In previous cases of calves with extensive carpal arthritis, the affected carpal bones were partially or completely removed before conducting arthrodesis (23, 25). Based on the preoperative imaging results in this case, the removal of the pathological femoral head could have been performed earlier, although the primary lesion was not determined. In preoperative planning to remove osseous lesions, 3D reconstruction of CTs has been used, as previously described (16). Additionally, for the preoperative planning of arthrotomy, contrast-enhanced 3D-CT may provide a useful vertical intra-articular view if arthrography is combined with CT (11).

FHO has previously been applied in the surgical treatment of femoral capital physeal fractures, coxofemoral luxation, acetabular fractures, and severe degenerative joint disease in bovine and equine cases (26–34). FHO is also applicable to hip arthritis, leading to infection control in 76–92% of patients (35, 36). However, its therapeutic efficacy is not well known as bovine hip arthritis has not previously been treated with FHO. In this case, it took 3 months to improve the weight-bearing lameness of the left hind limb, despite the final satisfactory result obtained from FHO. The delay in healing may be associated with insufficient resection around the femur during FHO. Girdlestone (35) recommended complete resection of the femoral head and neck at the level of the greater trochanter within the arthritic hip joints, a technique referred to as Girdlestone resection arthroplasty (35, 37). Later, FHO procedures were classified into four types based on postoperative radiographic evaluations of the resection regions: Type-1, resection of the femoral head at the level of the base of the femoral neck; Type-2, resection of the femoral head and neck while a small portion of the femoral neck was retained; Type-3, resection at the level of the intertrochanteric region; and Type-4, Girdlestone resection arthroplasty (38). Type-4 FHOs have been successfully applied to sick equines (31). Based on the postoperative CT scan in this case, our FHO procedure may have been equivalent to between Type-2 and Type-3. Extensive resection of the femoral head and neck can prevent postoperative recurrence due to the spread of infection from the remaining pathological bones (35, 38). The postoperative CT, in this case, indicated that the cut surface of the resected femur should have been extensively removed by more expanded debridement using a bone rongeur.

Additional postoperative CT evidence of FHO included close narrowing of the resected femoral surface into the acetabulum. This appears to indicate FHO-associated conditions of the hip joints, referred to as girdlestone pseudarthrosis (36). FHO-associated pseudarthrosis causes a decreased range of motion and shortened length of the treated hind limb in humans and hip pain in horses (33, 36). Conversely, a bovine case report suggested that pseudarthrosis may be favorable for relieving pain during ambulation (28). Satisfactory outcomes are commonly obtained when FHOs are performed on sick animals with body weights less than 100 kg (28–31). Additionally, FHOs effectively contribute to satisfactory outcomes in heavier, sick animals (32–34). However, improvements in weight-bearing lameness in treated animals usually occur several months after surgery (26, 29, 34). FHOs are also accompanied by postoperative complications such as septic arthritis, periarticular abscessation, fracture of the contralateral femur, and angular deformities of the ipsilateral and contralateral hind limbs (4, 27, 28, 32). Therefore, the combined use of imaging modalities during follow-up may help assessing the healing process, causes of delayed healing, postoperative complications, and postoperative judgments of surgical achievements soon after surgery (21).

The drainage method used in this case may have been a further cause of delayed healing. Drainage of synovial effusions has been effectively facilitated via a Penrose drain placed in arthritic joints after arthrotomy (23). However, the passive drainage obtained from a Penrose drain may have been insufficient for removing the effusions within the wide space of the hip joint (39), resulting in a delay in postsurgical recovery in this case. Additionally, the drainage effect of the Penrose drain might be limited because it has not been applied ventral to the surgical wound. The placement of a through-and-through drain system that facilitates both irrigation and drainage could lead to high therapeutic efficacy in bovine septic arthritis (6). However, the drainage technique might be difficult to perform for bovine hip arthritis, which commonly develops in deeper areas. Thus, hip arthritis should be controlled by postoperative drainage with a closed active drainage system, such as negative-pressure wound therapy using the syringe technique (39, 40).

## Conclusion

Hip arthritis can induce concurrent pathological changes that spread between the bones and soft tissues. Thus, hip arthritis can be routinely evaluated by the combined application of radiography and ultrasonography, which are superior at demonstrating bony and soft tissue lesions, respectively. CT should be used as a supplemental imaging modality to support radiography and ultrasonography because it can confirm the relationship between bony and soft tissue lesions.

## Data availability statement

The raw data supporting the conclusions of this article will be made available by the authors, without undue reservation.

## Ethics statement

The animal studies were approved by the Institutional Animal Care and Use Committee of Tottori University (approval number: 11-T-98). The studies were conducted in accordance with the local legislation and institutional requirements. Written informed consent was obtained from the owners for the participation of their animals in this study.

## Author contributions

TT: Conceptualization, Data curation, Investigation, Methodology, Writing – original draft, Writing – review & editing. YO: Investigation, Methodology, Writing – original draft. AN: Data curation, Investigation, Writing – original draft. YS: Data curation, Investigation, Writing – original draft. TM: Data curation, Investigation, Writing – original draft.
