# Supplementary material for: Case report: Imaging of septic arthritis in the hip joint of a calf treated with femoral head ostectomy
Source: Front Vet Sci. 2024 Jul 18;11:1292924. doi: 10.3389/fvets.2024.1292924 (PMC11291235; doi:10.3389/fvets.2024.1292924)
Supplement: Supplementary file 4 [file Data_Sheet_1.docx]

Supplementary Material

Imaging of septic arthritis in the hip joint of a calf treated with femoral head ostectomy

Takeshi Tsuka*, Yoshiharu Okamoto, Atsushi Nishiyama, Yuji Sunden, Takehito Morita

*** Correspondence:** Takeshi Tsuka: tsuka@tottori-u.ac.jp

**
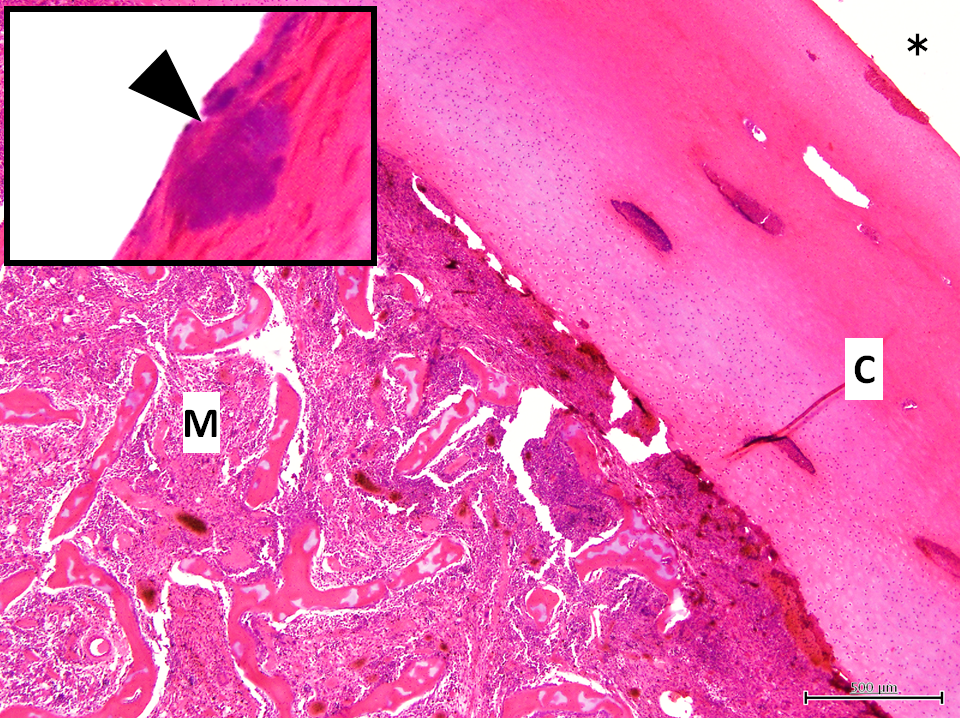
**

**Supplementary Figure 1.** Histopathological results of the resected femoral head. Aggregations of inflammatory cells and fibroblasts are evident in the bone marrow structure (M). Bacterial colonization (arrowhead) is evident in the bone cortex layers (C) at the lower-left inlet. Asterisk: joint cavity. HE staining. Bar = 500 μm.

**
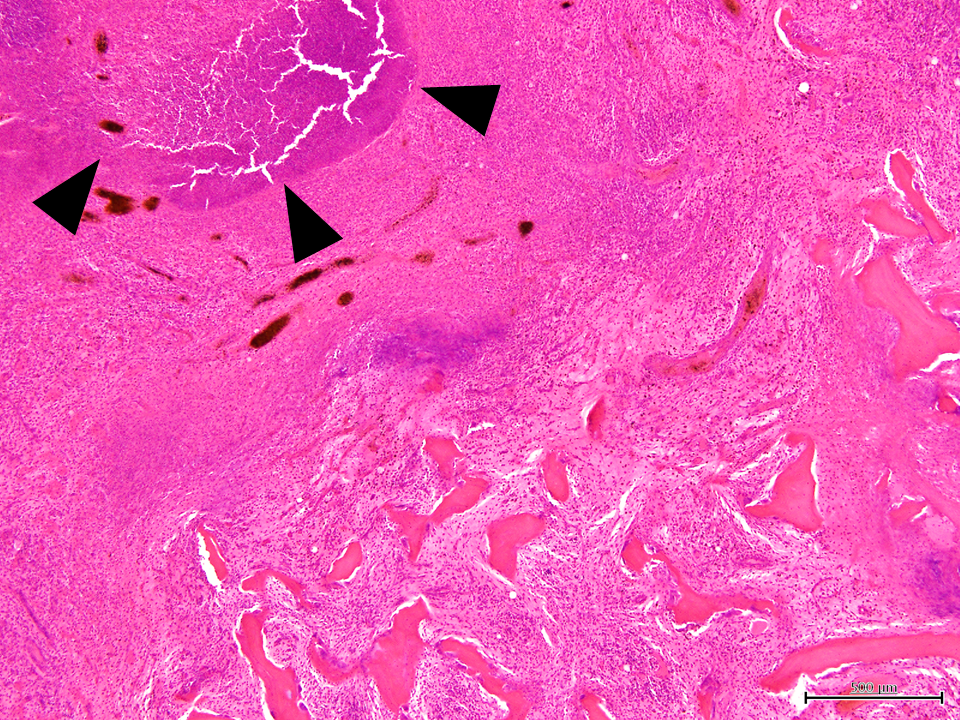
**

**Supplementary Figure 2.** Histopathological results of the resected femoral head. Small abscess foci (arrowheads) scattered in the pathological bone marrow tissues associated with osteomyelitis can be observed. HE staining. Bar = 500 μm.

**
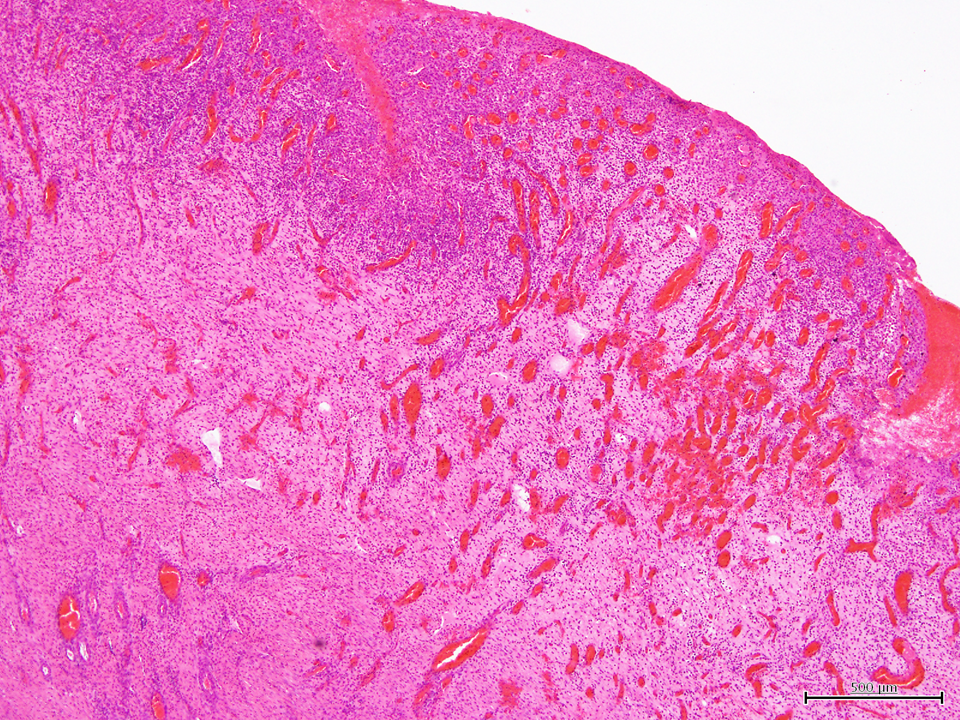
**

**Supplementary Figure 3.** Histopathological results of the lesion’s wall structure. Granulation tissues are predominantly seen together with rich neovasculature. HE staining. Bar = 500 μm.

**Supplementary movie 1**. The left panel shows an ultrasonogram scanning percutaneously between the greater trochanter and the wing of the ilium in the right hip joint. The right panel shows an ultrasonogram of the affected left hip joint. Between the beginning and 18 s of this video, ultrasonography near the pelvis (locations of the 1B transducer in Figure 3A) shows the flow of heterogeneous hyperechoic materials accompanying acoustic shadowing within the thick capsular wall. Between 19 s and the end of this video, the round, echogenic surface of the femoral head and neck is evident within the capsular lesion around the area of the femur (locations of the 1C transducer in Figure 3A) on the ultrasonogram.

**Supplementary movie 2.** After the capsular mass was excised through a 30-cm long skin incision, femoral head osteotomy for hip arthritis was performed according to the following surgical steps: (1) suction removal of the purulent fluids via a small cut hole made with cautery in the middle of the capsular wall; (2) removal of large caseous purulent materials; (3) irrigation with ozonated water within the mass’s cavity; and (4) resection of the femoral head using a bone chisel applied at the area of the femoral neck.

**Supplementary movie 3**. At initial admission, the affected calf showed severe weight-bearing lameness in the left hind limb (between the beginning and 34 s of this video). Five months after surgery, the animal was able to walk without lameness (between 35 s and the end of the video).
